# Treatment of Atrial Fibrillation in Patients with Dementia: A Cohort Study from the Swedish Dementia Registry

**DOI:** 10.3233/JAD-170575

**Published:** 2018-01-09

**Authors:** Ana Subic, Pavla Cermakova, Dorota Religa, Shuang Han, Mia von Euler, Ingemar Kåreholt, Kristina Johnell, Johan Fastbom, Liselia Bognandi, Bengt Winblad, Milica G. Kramberger, Maria Eriksdotter, Sara Garcia-Ptacek

**Affiliations:** a Department of Neurobiology, Care Sciences and Society, Division of Neurogeriatrics, Karolinska Institutet, Stockholm, Sweden; b Department of Neurology, University Medical Center, Ljubljana, Slovenia; c Medical Faculty, University of Ljubljana, Ljubljana, Slovenia; d National Institute of Mental Health, Klecany, Czech Republic; e Department of Neurobiology, Care Sciences and Society, Division of Clinical Geriatrics, Karolinska Institutet, Stockholm, Sweden; f Department of Geriatric Medicine, Karolinska University Hospital, Stockholm, Sweden; g Polish Academy of Sciences, Mossakowski Medical Research Center, Warsaw, Poland; h Department of Public Health Sciences, Karolinska Institutet, Stockholm, Sweden; i Department of Clinical Science and Education, Södersjukhuset, Stockholm, Sweden; j Department of Clinical Pharmacology, Karolinska University Hospital, Stockholm, Sweden; k Department of Medicine-Solna, Karolinska Institutet, Stockholm, Sweden; l Institute of Gerontology, School of Health and Welfare, Aging Research Network (ARN-J), Jönköping University, Jönköping, Sweden; m Aging Research Center, Karolinska Institutet and Stockholm University, Stockholm, Sweden; n Department of Internal Medicine, Neurology Section, Södersjukhuset, Stockholm, Sweden

**Keywords:** Atrial fibrillation, dementia, hemorrhage, ischemic stroke, warfarin

## Abstract

**Background:**

Patients with dementia might have higher risk for hemorrhagic complications with anticoagulant therapy prescribed for atrial fibrillation (AF).

**Objective:**

This study assesses the risks and benefits of warfarin, antiplatelets, and no treatment in patients with dementia and AF.

**Methods:**

Of 49,792 patients registered in the Swedish Dementia Registry 2007–2014, 8,096 (16%) had a previous diagnosis of AF. Cox proportional hazards models were used to calculate the risk for ischemic stroke (IS), nontraumatic intracranial hemorrhage, any-cause hemorrhage, and death.

**Results:**

Out of the 8,096 dementia patients with AF, 2,143 (26%) received warfarin treatment, 2,975 (37%) antiplatelet treatment, and 2,978 (37%) had no antithrombotic treatment at the time of dementia diagnosis. Patients on warfarin had fewer IS than those without treatment (5.2% versus 8.7%; *p* < 0.001) with no differences compared to antiplatelets. In adjusted analyses, warfarin was associated with a lower risk for IS (HR 0.76, CI 0.59–0.98), while antiplatelets were associated with increased risk (HR 1.25, CI 1.01–1.54) compared to no treatment. For any-cause hemorrhage, there was a higher risk with warfarin (HR 1.28, CI 1.03–1.59) compared to antiplatelets. Warfarin and antiplatelets were associated with a lower risk for death compared to no treatment.

**Conclusions:**

Warfarin treatment in Swedish patients with dementia is associated with lower risk of IS and mortality, and a small increase in any-cause hemorrhage. This study supports the use of warfarin in appropriate cases in patients with dementia. The low percentage of patients on warfarin treatment indicates that further gains in stroke prevention are possible.

## INTRODUCTION

Atrial fibrillation (AF) is the most common cardiac arrhythmia and its prevalence increases with age appearing in 10% of those older than 80 years [1]. AF is responsible for about 20% of all ischemic strokes (IS) [2]. The risk of stroke is increased in patients with dementia, [3] representing an important cause of morbidity and death [4, 5]. Oral anticoagulant (OAC) therapy, traditionally warfarin, is used to prevent stroke in patients with AF. Previous studies have shown that adjusted-dose warfarin is more effective than aspirin at reducing IS, but increases bleeding complications [6–8]. However, patients with dementia have often been excluded from studies establishing the benefit of warfarin and there are still few studies on stroke prevention and treatment in this population [9].

Warfarin may be underused or inconsistently prescribed in older patients with dementia [10]. Old age and dementia are associated with greater risk for hemorrhagic complications through different mechanisms, including falls, and polypharmacy leading to interactions with warfarin metabolism. Leukoaraiosis and cerebral amyloid angiopathy have been associated with higher risk for intracranial hemorrhage (ICH) [9]. Similar mechanisms may also explain the suggested increased ICH rate for aspirin treatment in patients with dementia [11].

For these reasons, the American Academy of Neurology guidelines for stroke prevention in AF state that data in patients with AF who have moderate to severe dementia are insufficient to determine whether anticoagulants are safe [12]. European Stroke Organisation guidelines do not specifically contraindicate OAC in dementia patients, but do not recommend them in patients with co-morbid conditions such as falls or poor compliance [13]. The European Society of Cardiology guidelines do not consider dementia to be a contraindication for anticoagulation unless compliance cannot be ensured [14].

The present cohort study aims to assess the risks and benefits of OAC and antiplatelet use for stroke prevention in AF in patients with dementia, and evaluate the association between treatment and risk of ischemic and hemorrhagic stroke, any-cause hemorrhage, and death.

## METHODS

We performed a longitudinal cohort study on patients registered in the Swedish Dementia Registry (SveDem). Information on AF, treatment, and comorbidities was obtained from the Swedish Patient Register and the Swedish Prescribed Drug Register. Data on death was obtained from the Swedish Population Register.

This study complies with the Declaration of Helsinki and was approved by the regional ethical review board in Stockholm, Sweden. Patients were informed of registration in SveDem and could decline participation. Data were de-identified before analysis.

### Study population

SveDem is a national registry that aims to improve the quality of diagnostic workup, treatment, and care of patients with dementia in Sweden. The registration process and variables in SveDem have been previously described [15, 16]. Briefly, patients with newly diagnosed dementia are registered by physicians in specialist or primary care together with demographic and treatment variables. For this study, the diagnoses were classified as Alzheimer’s dementia (AD), vascular dementia, mixed Alzheimer’s and vascular dementia, or other dementia disorders. Information on age, sex, Mini-Mental State Examination (MMSE) score, and living conditions was obtained from SveDem at the time of dementia diagnosis.

Information about co-morbidities occurring from 1998 until the end of 2014 coded according to International Classification of Diseases 10 (ICD-10) [17] was collected from the Swedish Patient Register. This register covers inpatient and outpatient specialist care encounters in Sweden [18]. Patients with AF were identified by the code I48 present either as main or contributory diagnosis at any point between the year 1998 and the date of dementia diagnosis. Other comorbidities identified as either main or contributory diagnosis from 1998 until the time of dementia diagnosis were diabetes mellitus (E10-E13), hypertension (I10-I15), liver diseases (K70-77), kidney diseases (N10-19), heart failure (I50), presence of cardiac pacemaker (Z950, DF016, DF014, FPE, FPF, FPG, FPJ, DZXG40, or ZXG50), and ischemic heart disease (I20-I25).

Furthermore, previous main diagnoses of stroke of any type (I64), IS (I63), nontraumatic ICH (I60-I62), and traumatic ICH (S063-S068) from 1998 until the time of dementia diagnosis were identified. A composite variable of main diagnoses of systemic and intracranial hemorrhage, both traumatic and spontaneous was considered (any-cause hemorrhage). Any-cause hemorrhage included traumatic ICH (S063-S068); nontraumatic ICH (I60-I62); hemorrhage from respiratory passages (R04); anemia (D50 and D62); hemorrhage unspecified (R58); gastrointestinal bleeding (K92); and traumatic shock (T794). For outcomes, we used a record of the disease occurring as main diagnosis after the date of dementia diagnosis: IS (ICD-10 I63), nontraumatic ICH (ICD-10 I60-I62), any-cause hemorrhage (described above), and death (obtained from the Swedish Death Register).

Drug use 2005–2014 was obtained from the Swedish Prescribed Drug Register, which includes all prescription-drugs dispensed to a patient in any pharmacy in Sweden [19]. For each individual, a medication list at the date of measure was constructed according to the filled prescriptions registered in the Swedish Prescribed Drug Register during the three month period preceding the date of dementia diagnosis.

Drugs were coded according to the Anatomical Therapeutic Chemical classification system: acetylsalicylic acid (B01AC06, N02BA01, N02BA51), warfarin (B01AA03), and clopidogrel (B01AC04). Patients treated with novel oral anticoagulants (NOACs) were excluded because these treatments were uncommon in Sweden during the study period. The following groups of treatment were considered: 1) patients receiving only warfarin treatment at the time of dementia diagnosis (patients with coexisting antiplatelet treatment were excluded), 2) patients with only antiplatelet treatment (receiving only aspirin, only clopidogrel or combination of aspirin with clopidogrel), and 3) patients without any antiplatelet or anticoagulant treatment. Drug prescription was decided by their physician and was not randomized. The number of habitual drugs taken at the time of dementia diagnosis was used as a proxy for comorbidity, calculated after excluding warfarin and antiplatelets [16, 20].

### Statistical analyses

**Fig.1:**
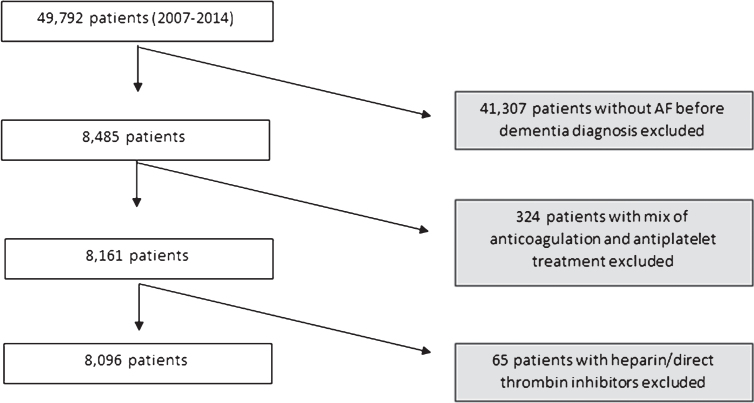
Selection of the study sample.

From 49,792 patients, 8,485 (17%) had a diagnosis of AF. After exclusion of patients who used both antiplatelets and warfarin or other kinds of anticoagulants, the final study sample of patients with dementia and AF was 8,096 (Fig. 1). Baseline characteristics of the patients at the time of dementia diagnosis and the outcomes during follow-up were compared between treatment groups using chi-square test or *T*-test. Descriptives are shown as means±standard deviation (SD), median and interquartile range (IQR), or frequency (number—n and percentage–%), as appropriate.

Survival analyses to identify factors associated with risk for IS, ICH, any-cause hemorrhage, and death were performed using Cox proportional hazards regression models to estimate hazard ratios (HR) with 95% confidence intervals (CI). The proportionality of hazards was checked using visual procedures. Models adjusted for age and sex were performed. Subjects with observation time <1 day were excluded (*n* = 6). The final adjusted models were arrived at by testing any variables that presented baseline differences between the groups with *p* < 0.25 in univariate comparisons and other variables previously shown to affect mortality in Swedish patients with dementia [16]: variables were then kept in the survival models if they were significant and/or substantially improved the model. The final models were adjusted for age, sex, number of medication, MMSE, dementia type (AD versus others), nursing home placement, and previous diagnosis of diabetes, hypertension, heart failure, IS, any-cause hemorrhage, liver diseases, and kidney diseases. Models were repeated for all four outcomes: IS, ICH, any-cause hemorrhage, and death. Adjusted and unadjusted models for all four outcomes were repeated with stratification by sex, previous pacemaker, previous IS, and previous any-cause hemorrhage. We performed *post-hoc* propensity score adjusted models. These were obtained from multiple logistic regression including the variables age, sex, number of medication, MMSE, dementia type (AD versus others), nursing home placement, previous diagnosis of diabetes, hypertension, heart failure, pacemaker IS, any-cause hemorrhage, anemia, gastrointestinal bleeding, hip fracture, liver diseases, and kidney diseases. The interactions between number of medications and liver or kidney diseases were tested. Two linear splines with a node at 79 years (sample median) were used for age, while MMSE and number of drugs were entered as linear variables. Comorbidities were entered as dichotomous variables as described above.

Two tailed *p* < 0.05 was considered to be statistically significant in all analytical procedures. Analyses were performed using the Statistical Package for the Social Sciences software version 22 (IBM Corporation, Armonk, NY, USA) and STATA® version 12.1 (StataCorp, College Station, TX, USA).

## RESULTS

This study included 8,096 patients with dementia and AF (82 years old; 52% women; Table 1). At the time of dementia diagnosis, 2,143 (26%) patients received warfarin, 2,975 (37%) antiplatelets, and 2,978 (37%) neither antiplatelet nor anticoagulant treatment. Patients on warfarin were younger (81 years versus 83 antiplatelet versus 82 years no treatment, *p* < 0.001), less frequently women (48% versus 52% in the antiplatelet group and 55% in no-treatment groups; *p* < 0.001), and had higher MMSE (22 versus 21 patients with antiplatelets or without treatment; *p* < 0.001). Patients were followed up for a median of 636 days (IQR 805) until death or the end of 2014.

**Table 1:** Baseline characteristics of study population

AF present at the time of dementia diagnosis (*N* = 8,096)	Patients without treatment (*n* = 2,978; 37%)	Patients treated with warfarin (*n* = 2,143; 27%)	*p*-value for dif. to no treatment	Patients with antiplatelet treatment (*n* = 2,975; 37%)	*p*-value for dif. to warfarin
Age at diagnosis (mean±SD)	82.3±6.5	80.8±5.8	<0.001	83.3±6.2	<0.001
Female gender, *n* (%)	1544 (51.8)	1028 (48.0)	0.006	1627 (54.7)	<0.001
MMSE, med (IQR)	21 (29)	22 (30)	<0.001	21 (28)	<0.001
Type of dementia
AD, *n* (%)	582 (19.5)	462 (21.6)	0.077	555 (18.7)	0.010
MD, *n* (%)	662 (22.2)	433 (20.2)	0.081	661 (22.2)	0.083
VaD, *n* (%)	801 (26.9)	630 (29.4)	0.049	811 (27.3)	0.093
OD, *n* (%)	933 (31.3)	618 (28.8)	0.056	948 (31.9)	0.020
Living alone, *n* (%)	1309 (48.4)	793 (39.4)	<0.001	1499 (58.2)	<0.001
Living in an institution, *n* (%)	458 (15.4)	155 (7.2%)	<0.001	565 (19.1)	<0.001

AF, atrial fibrillation, *n* (%), number of patients in each category and percentage relative to the whole cohort; SD, standard deviation; IQR, interquartile range, MMSE, Mini-Mental State Examination, AD, Alzheimer’s dementia; VaD, vascular dementia; MD, mixed AD and VaD; OD, other dementia diagnoses including Parkinson’s disease with dementia, dementia with Lewy bodies, frontotemporal dementia, and unspecified dementia. Missing values: Age at diagnosis, dementia type and sex had no missing values. MMSE had 422 (5.2%) missing values, living alone 803 (9.9%), living in institution 27 (0.3%).

During the follow-up, 111 (5.2%) IS occurred in the warfarin group, compared to 180 (6.0%) in the antiplatelet (*p* = 0.187) and 260 (8.7%) in the no-treatment group (*p* < 0.001). ICH appeared in 1.9% of patients on warfarin compared to 1.6% on antiplatelets (*p* = 0.553) and 1.3% with no treatment (*p* = 0.136). There were no significant differences in the rates of any-cause hemorrhage. During follow-up, 3,094 deaths occurred: in the warfarin group 600 (28%) died, compared to 1,389 (46.7%) in the antiplatelet, and 1,105 (37.1%) in the no-treatment groups (*p* < 0.001). Comorbidities of the patients are listed on Table 2.

**Table 2:** Comorbidities in patients with atrial fibrillation before and after dementia diagnosis

Atrial fibrillation (*N* = 8,096)	No treatment (*n* = 2,978; 37%)	Warfarin (*n* = 2,143; 27%)	*p*-value for dif. to no treatment	Antiplatelets (*n* = 2,975; 37%)	*p*-value for dif. to warfarin
Number of drugs, med (IQR)	5.0 (23)	6.0 (21)	<0.001	6.0 (23)	<0.001
Comorbidities at dementia diagnosis
Diabetes mellitus, *n* (%)	520 (17.5)	460 (21.5)	<0.001	596 (20.0)	0.212
Hypertension, *n* (%)	1817 (61.0)	1400 (65.3)	0.002	1908 (64.1)	0.378
Liver disease, *n* (%)	50 (1.7)	20 (0.9)	0.023	34 (1.1)	0.469
Kidney disease, *n* (%)	334 (11.2)	179 (8.4)	0.001	327 (11.0)	0.002
Heart failure, *n* (%)	1062 (35.7)	841 (39.2)	0.009	1100 (37.0)	0.099
Pacemaker, *n* (%)	388 (13)	351 (16.4)	0.001	291 (9.8)	<0.001
Previous ischemic heart disease, *n* (%)	985 (33.1)	750 (35.0)	0.152	1202 (40.4)	<0.001
Previous all stroke, *n* (%)	659 (22.4)	503 (23.5)	0.258	704 (23.7)	0.873
Previous ischemic stroke, *n* (%)	494 (16.6)	432 (20.2)	0.001	533 (17.9)	0.043
Previous nontraumatic intracranial hemorrhage, *n* (%)	116 (3.9)	23 (1.1)	<0.001	97 (3.3)	<0.001
Previous traumatic intracranial hemorrhage, *n* (%)	81 (2.7)	22 (1.0)	<0.001	65 (2.2)	0.002
Previous any hemorrhage, *n* (%)	571 (19.2)	284 (13.3)	<0.001	467 (15.7)	0.015
Outcomes after dementia diagnosis
All stroke, *n* (%)	243 (8.2)	176 (8.2)	0.946	338 (11.4)	<0.001
Ischemic stroke, *n* (%)	180 (6.0)	111 (5.2)	0.187	260 (8.7)	<0.001
Nontraumatic intracranial hemorrhage, *n* (%)	40 (1.3)	40 (1.9)	0.136	49 (1.6)	0.553
Traumaticintracranial hemorrhage, *n* (%)	32 (1.1)	23 (1.1)	0.996	35 (1.2)	0.731
Any hemorrhage, *n* (%)	199 (6.7)	160 (7.5)	0.278	205 (6.9)	0.430
Death, *n* (%)	1105 (37.1)	600 (28.0)	<0.001	1389 (46.7)	<0.001

AF, atrial fibrillation, N (%), number of patients in each category and percentage relative to the whole cohort, IQR, interquartile range. For number of drugs there were 339 (4.2%) missing.

**Table 3:** Hazard ratios (HR) of ischemic stroke, nontraumatic intracranial hemorrhage, any hemorrhage and death compared to no treatment

	HR for ischemic stroke (95% CI)	HR for nontraumatic intracranial hemorrhage (95% CI)	HR for any hemorrhage (95% CI)	HR for death (95% CI)
No treatment	Ref.	Ref.	Ref.	Ref.
Warfarin	0.76 (0.59–0.98)*	1.47 (0.91–2.37)	1.08 (0.87–1.35)	0.84 (0.59–0.98)**
Antiplatelets	1.25 (1.01–1.54)*	1.29 (0.81–2.04)	0.84 (0.68–1.04)	0.91 (0.83–0.99)*

Hazard ratios (HR) and 95% confidence intervals (CI) for the association of treatment with warfarin or antiplatelets (aspirin or clopidogrel) and risk of ischemic or nontraumatic intracranial hemorrhage and death after dementia diagnosis, as obtained from Cox Hazards regression models. Adjusted for age, sex, number of drugs, Mini-Mental State Examination, dementia type (Alzheimer’s dementia versus other), nursing home placement, previous diabetes, hypertension, heart failure, ischemic stroke, any-cause hemorrhage, liver and kidney disease.

**Table 4:** Hazard ratios of ischemic stroke, nontraumatic intracranial hemorrhage, any hemorrhage and death compared to antiplatelet treatment

	HR for ischemic stroke (95% CI)	HR for nontraumatic intracranial hemorrhage (95% CI)	HR for any hemorrhage (95% CI)	HR for death (95% CI)
Antiplatelets	Ref.	Ref.	Ref.	Ref.
Warfarin	0.62 (0.49–0.78)***	1.16 (0.75–1.80)	1.28 (1.03–1.59)*	0.93 (0.84–1.03)
No treatment	0.80 (0.65–0.99)*	0.81 (0.51–1.28)	1.19 (0.96–1.47)	1.12 (1.02–1.23)*

Same analyses as presented in Table 3, but with antiplatelets as reference category. Adjusted for age, sex, number of drugs, Mini-Mental State Examination, dementia type (Alzheimer’s dementia versus other), nursing home placement, previous diabetes, hypertension, heart failure, ischemic stroke, any-cause hemorrhage, liver and kidney disease.

### Survival analyses

Compared to no treatment, in the fully-adjusted model, warfarin was associated with a lower risk for IS (HR 0.76, 95% CI 0.59–0.98), while antiplatelets were associated with increased risk (HR 1.25, 95% CI 1.01–1.54). In sex-stratified analyses, warfarin was associated with reduced risk of IS compared to no treatment in women (HR 0.65, 95% CI 0.45–0.94), with non-significant results in men (HR 0.89, 95% CI 0.62–1.27).

There were no significant associations for ICH in patients on warfarin compared to no treatment (HR 1.47, 95% CI 0.91–2.37) or antiplatelets (HR 1.16, 95% CI 0.75–1.80). No significant difference for ICH was found between antiplatelets and no treatment (HR 1.29, 95% CI 0.81–2.04).

Similarly, there was no significant association for any-cause hemorrhage between warfarin or antiplatelets compared to no treatment (warfarin HR 1.08, 95% CI 0.87–1.35; antiplatelets HR 0.85, 95% CI 0.68–1.04). However, compared to antiplatelets there was a higher risk of hemorrhage with warfarin (HR 1.28, 95% CI 1.03–1.59). Stratifying by sex, the association between any-cause hemorrhage and warfarin was shown only in men (HR 1.43, 95% CI 1.05–1.96). In men, there was an association with lower risk for any-cause hemorrhage in patients on antiplatelets compared to no treatment (HR 0.71, 95% CI 0.52–0.97).

Treatment with warfarin or antiplatelets was associated with lower HR for death compared to no treatment (warfarin: HR 0.84, 95% CI 0.59–0.98, antiplatelets: HR 0.91, 95% CI 0.83–0.99). However, in propensity score adjusted models, treatment with antiplatelets was not associated with lower risk for death compared to no treatment (HR 0.99, 95% CI 0.90–1.08) (Supplementary Table 1). In patients without previous IS, there was an association between warfarin and lower HR for death compared to no-treatment (HR 0.84, 95% CI 0.74–0.95). In patients without previous hemorrhage there was lower HR for death in patients with warfarin compared to no treatment (HR 0.84, 95% CI 0.75–0.95), which was also present in the antiplatelet group (HR 0.90, 95% CI 0.81–1.00). Hazard ratio analysis is presented in Tables 3 and 4.

## DISCUSSION

In this Swedish cohort of dementia patients with AF, warfarin treatment at the time of dementia diagnosis was associated with lower risk of IS than antiplatelets or no treatment. This is in line with the protective effect of warfarin for stroke in patients with AF observed in the general [6–8, 21] and older [22] populations, as well as in patients with recently diagnosed dementia [23].

In our study, rates of IS were in the range from 5.2% in the warfarin group, 6.0% in the antiplatelet to 8.7% in the no-treatment group, which would correspond to annual rates 3% in warfarin group, 3.4% in the antiplatelet, and 5.0% in the no-treatment group, which is relatively low compared to other studies. The average IS rate in the general AF population (mean age 71 years) was 4.6% for warfarin for secondary prevention and 1.8% per year for primary prevention in previous studies [6]. With antiplatelet treatment this rises to 10.5% for secondary and 3.9% for primary prevention compared to 13.0% per year for secondary prevention and 4.1% per year for primary prevention in untreated participants [6]. However, in patients with dementia there may be a general underdiagnosis of stroke, if cognitive symptoms obscure new neurological focality or if patients in palliative stages are not sent to hospital. This could explain lower annual rates of stroke found in our study.

The most common adverse effects of warfarin and antiplatelets are hemorrhages, and most studies on primary and secondary stroke prevention in AF show increase in absolute risk for ICH and major bleeding [6–8, 21]. In our study the crude rates of hemorrhagic stroke and any-cause hemorrhage did not differ between the treatment groups, although in adjusted survival analyses an association was found between warfarin and risk of any-cause hemorrhage compared to antiplatelet treatment. In a previous study on an older population with AF, no significant differences were found in the rate of ICH or major hemorrhage with warfarin compared to aspirin [22], and another smaller study among octogenarians did not detect major bleedings in any of the treatment groups [24]. Lower incidence of ICH with warfarin in these studies can be partly explained by patient selection: in the Birmingham Atrial Fibrillation Treatment of the Aged Study patients for whom warfarin was clearly indicated were excluded because of ethical reasons, so participation in study was restricted to patients with lower risk for stroke, for whom there was clinical uncertainty which of the two treatments should be used [22]. Additionally, pooled data from five randomized trials indicated almost no increase in frequency of major bleeding with antithrombotic treatment (1.3% for the warfarin group, 1.0% for the aspirin group, and 1.0% for the control group) [25]. A meta-analysis on twenty-nine randomized controlled trials investigating long-term antithrombotic therapy in patients with AF found that the risk for ICH doubled with warfarin compared to aspirin, although the absolute risk increase was small (0.2% /year) [6]. There was a similar absolute rate increase for major extracranial bleeding events for warfarin versus control (3%), warfarin versus aspirin (2%), and for aspirin versus control (2%) [6]. These studies were conducted among older patients, with a mean age of 71 years.

In the European/Australasian Stroke Prevention in Reversible Ischaemia Trial, which investigated secondary prevention of stroke and compared OAC with a target international normalized ratio (INR) between 2.0–3.0 with aspirin treatment, the annual incidence of ICH was between 0.31–1.21% in anticoagulated patients [26]. The incidence of ICH for aspirin was 0.39% per year [27]. This compares with the incidence of ICH in the present study which ranges from 1.3 to 1.9% over a follow-up which is approximately 1.7 years, which translates approximately into 1.12% ICH per year in dementia patients treated with warfarin. Our findings suggest that the hemorrhagic risk in patients with dementia is similar to what has previously been reported among older patients. When evaluating warfarin prescription, the risk of IS must be weighed against the risk of ICH and other hemorrhagic complications. In our data, the absolute rate of IS during follow-up was approximately 3–5 times higher than that for ICH, which is comparable to other studies [28].

In our cohort, there was an association with increased risk for ICH with advanced age, previous hypertension, and previous hemorrhage. Previous studies that investigated use of warfarin for prevention of thromboembolism in AF showed higher rate of bleeding in the older age groups, but also higher rate of IS indicating a greater need for anticoagulation [29, 30]. However, in previous studies older patients (aged > 75) were significantly underrepresented [9]. In many of these studies, it was also shown that occurrence of ICH is mainly related to the intensity of anticoagulant therapy. For example, in the Stroke Prevention in Reversible Ischaemia Trial, the incidence of major hemorrhages in patients with INR between 2.0–3.0 was two thirds lower than in those with INR 3.0–4.5 [27].

For this reason, maintaining an INR within the target range is critical to treatment success and nonadherence or drug-drug interactions that threaten this balance are sufficient reasons to withhold or withdraw treatment [13]. Dementia has been shown to be one of the main risk factors for nonadherence to OAC therapy [31]. In a recent study on cognitive function and adherence to anticoagulation treatment, 46.8% of patients with cognitive disorders had low levels of medication adherence [32]. However, another study in patients with AF who received a new diagnosis of dementia has shown good anticoagulation control, as measured according to time in therapeutic range (TTR) (mean TTR before dementia diagnosis was 58.8±22.6% for all patients on warfarin, 62.0±18.4% for those who remained on warfarin after diagnosis with dementia and 65.8±18.9% for those without dementia) [23].

In our study, a high proportion of patients with dementia and AF were living alone (40% with warfarin treatment, 48% with antiplatelets, and 58% with no treatment). Monitoring and care of patients with AF in Sweden is well organized, with many patients receiving help with medication administration and INR control in their own homes—and this must be kept in mind before generalizing the findings of this study to other countries [33]. Cardiovascular medication use in patients with dementia shows regional variations, which must be taken into account when extrapolating these results to other cohorts [34]. Warfarin and antiplatelet treatment were associated with lower risk for death compared to no treatment in the present study. Compared to antiplatelet treatment, there was a non-significant trend toward lower risk for death with warfarin. Some previous studies reported better survival with warfarin compared to placebo [6, 35], while others comparing warfarin to antiplatelet therapy found no significant differences in survival [8, 21]. Pooled data from twenty-nine randomized controlled trials showed lower all-cause mortality for warfarin versus controls (RRR, relative risk reduction, 26%), for warfarin versus aspirin (RRR, 9%), and for aspirin versus controls (RRR, 14%) [6]. Warfarin was associated with lower mortality (HR 0.72, 95% CI 0.60–0.87, *p* < 0.001) also in patients with dementia [23]. This effect has to be interpreted with caution, because there can be bias occurring if patients with a lower life expectancy were not treated at all. In our study, when stratifying by sex, treatment with warfarin compared to no treatment was associated with reduced risk of IS in women, while results in men were not significant. This finding is in line with previous studies showing that women with AF have an increased risk of IS, [36] and would therefore benefit more from warfarin.

The current study has several limitations. The data has been obtained from registries, and important factors affecting treatment decisions, such as possible contraindications for warfarin treatment, patient preferences, compliance, INR at the time of occurrence of the adverse events and TTR, were not available. Patients were classified according to their treatment status at the time of dementia diagnosis. Therefore, patients could have changed treatment groups in the course of follow-up. These changes could possibly have led to the underestimation of the protective effect of warfarin on stroke risk, but also an underestimation of the hemorrhagic complications, if as expected, patients are taken off warfarin as cognitive deterioration progresses. The Patient Registry covers all inhospital and specialist clinic diagnosis: since acute stroke is primarily a hospital diagnosis, the coverage for this outcome should be good, although diagnostic precision is always a concern. The estimated coverage of SveDem is approximately 35% [15]. There are no studies on the differences between dementia patients in SveDem and non-registered patients, however, we can assume that patients included in the quality register are in general healthier [37]. This may bias the generalizability of the results towards a healthier group of patients and may lead to an underestimation of the comorbidities and their influence on risk of stroke and death.

This is an observational study and treatment was not randomly allocated, so there may be important differences between patients in the different treatment groups. Antithrombotic treatment is often withheld in patients with poor survival prognosis. The data to assess frailty index and information on number of falls was not available and controlling for comorbidities and cognitive level may not adequately account for this. The low incidence of ICH and any hemorrhage in the warfarin group in this study could be partly explained because possible contraindications for warfarin were taken into consideration when prescribing the medication, which could have eliminated differences between the treatment groups. The excess risk for IS found with antiplatelets could indicate that this group of patients were prescribed antiplatelets for reasons other than AF (confounding by indication), which would imply a higher risk for atherothrombotic stroke, in addition to the embolic stroke risk. Confounding by indication could also explain the lower risk of ICH in men taking antiplatelets, compared to no treatment. For outcomes, we included only IS without peripheral emboli, which can be also caused by AF, although much rarer and often clinically silent [38]. This study does not include the new generation of oral anticoagulants because their use has only recently increased in Sweden and the number of patients and follow-up time during the study period was limited.

One of the strengths of this study is the inclusion of a uniquely large sample of patients with dementia from a nationwide register. Moreover, the diagnoses of AF, comorbidities and information on drugs were obtained from Swedish health registers that have a complete national coverage. The diagnosis of AF and comorbidities were obtained from Swedish National Patient register, which has high overall diagnostic validity (85–95%) [18]. Monitoring of the data in SveDem is performed by random cross-checks of histories and entries [15] and around 5% of dementia diagnoses change during the first year of follow-up [16]. Despite the irruption of NOACs, warfarin remains the most prescribed anticoagulant worldwide and studies examining the risk-benefits of warfarin treatment among patients with dementia are still clinically relevant. Since randomized studies in this subject are ethically and methodologically challenging, cohort studies represent the next-best option in answering these questions. The low percentage of treated patients is also a surprising finding, and implies that great gains can be made in stroke prevention in patients with dementia and AF in Sweden by extending treatment to all appropriate cases. However, as treatment is extended to borderline cases, the risk of complications would increase and future cohort studies should monitor trends in treatment, complications, and the effects of the introduction of NOACs among patients with dementia in Sweden.

### Conclusions

In this nationwide cohort study of patients with dementia and AF, the use of warfarin compared to no treatment was associated with lower risk of ischemic stroke and mortality. The use of warfarin compared to antiplatelets was associated with lower risk of ischemic stroke. There was no significant increase in hemorrhagic complications with warfarin compared to no treatment, and no differences in nontraumatic ICH. A higher risk of any-cause hemorrhage was shown with warfarin compared to antiplatelet treatment but the absolute risk was small. The crude rates of IS were 3–5 times higher than the rates of ICH, which is in line with previous studies. This supports the use of warfarin in appropriate cases in patients with dementia, and indicates that current patient selection and warfarin control in Sweden have succeeded in avoiding excess hemorrhagic complications.

## Supplementary Material

Supplementary Material
