# Supplementary material for: Treatment of Atrial Fibrillation in Patients with Dementia: A Cohort Study from the Swedish Dementia Registry
Source: J Alzheimers Dis. 2018 Jan 9;61(3):1119–28. doi: 10.3233/JAD-170575 (PMC5798527; doi:10.3233/JAD-170575)
Supplement: Supplementary Material [file jad-61-jad170575-s001.docx]

**Supplementary Material**

Number needed to treat (NNT) for patients on warfarin comparing with those on aspirin to prevent IS was 28.6. Number needed to harm (NNH) for warfarin comparing with aspirin regarding ICH outcome, was 333.3. NNH for any hemorrhage was 166.7, comparing patients with warfarin to aspirin group. NNT for patients on warfarin comparing with no treatment group to prevent IS was 125.0. NNH for warfarin comparing with no treatment regarding ICH outcome, was 166.7. NNH for any hemorrhage was 125.0, comparing patients with warfarin to those with no treatment.

**Supplementary Table 1.** Propensity scores for ischemic stroke, nontraumatic intracranial hemorrhage, any hemorrhage, and death compared to no treatment.

|  | **HR for ischemic stroke (95% CI)** | **HR for nontraumatic intracranial hemorrhage (95% CI)** | **HR for any hemorrhage (95% CI)** | **HR for death (95% CI)** |
| --- | --- | --- | --- | --- |
| **No treatment** | Ref. | Ref. | Ref. | Ref. |
| **Warfarin** | 0.76 (0.59-0.98)* | 1.47 (0.91-2.37) | 1.08 (0.86-1.34) | 0.88 (0.79-0.98)* |
| **Antiplatelets** | 1.23 (1.00-1.51)* | 1.21 (0.77-1.90) | 0.84 (0.68-1.03) | 0.99 (0.90-1.08) |

Propensity scores were obtained from multiple logistic regression including the variables age, sex, number of medication, MMSE, dementia type (Alzheimer’s dementia versus others), nursing home placement, previous diagnosis of diabetes, hypertension, heart failure, pacemaker, ischemic stroke, any-cause hemorrhage, anemia, gastrointestinal bleeding, hip fracture, liver diseases and kidney diseases. Hazard ratios (HR) and 95% confidence intervals (CI) for the association of treatment with warfarin or antiplatelets (aspirin or clopidogrel) and risk of ischemic or nontraumatic intracranial hemorrhage and death after dementia diagnosis are presented.
